# A numerical control machining tool path step error prediction method based on BP neural network

**DOI:** 10.1038/s41598-023-43617-6

**Published:** 2023-09-28

**Authors:** Zi-Yu Zhang, Wei Liu, Peng-Fei Li, Jia-Ping Zhang, Lv-Yang Fan

**Affiliations:** College of Mechanical Engineering, Suzhou University of Science and Technology, Suzhou, 215000 China

**Keywords:** Mechanical engineering, Aerospace engineering

## Abstract

Step error calculation of numerical control (NC) machining tool path is a premise for generating high-quality tool path and promoting its application. At present, iterative methods are generally used to calculate step error, and the computation time increases when accuracy improves. Neural networks can be calculated on GPUs and cloud platforms, which is conducive to reducing computation time and improving accuracy through continuous learning. This article innovatively introduces a BP neural network model to predict step error values. Firstly, the core parameters required for step error calculation are taken as the data samples to construct the neural network model, and map to the same scale through Z-score normalization to eliminate the adverse effects of singular parameters on the calculation results. Then, considering only a small number of parameters determine theoretical values of step error, the Dropout technique can drop hidden layer neurons with a certain probability, which is helpful to avoid overfitting and used in the neural network model design. In the neural network model training, this paper adds the Stochastic Gradient Descent with Momentum (SGDM) optimizer to the back propagation of network training in order to improves the network’ stability and accuracy. The proposed neural network predicts step error of samples from three surface models, the results show that the prediction error decreases as sample training increases. After trained by 15% of the surface samples, the neural network predicts the step errors of the remaining samples. Compared with theoretical values, more than 99% of the predicted values have an absolute error less than 1 μm. Moreover, the cost time is only one-third of the geometric method, which verifies the effectiveness and efficiency of our method.

## Introduction

In NC machining, when a tool moves from one cutter location (CL) point to the next one in the feed direction, the tool envelope surface is generated. The step error of two adjacent CL points is the maximum error between the tool envelope and the cutter contacting (CC) curve. In high-precision surface machining, step error of tool path is prohibited greater than the allowable maximum value. So, step error must be calculated and checked in the process of tool path generation.

### Geometry principle and calculation methods of step error

**Figure 1:**
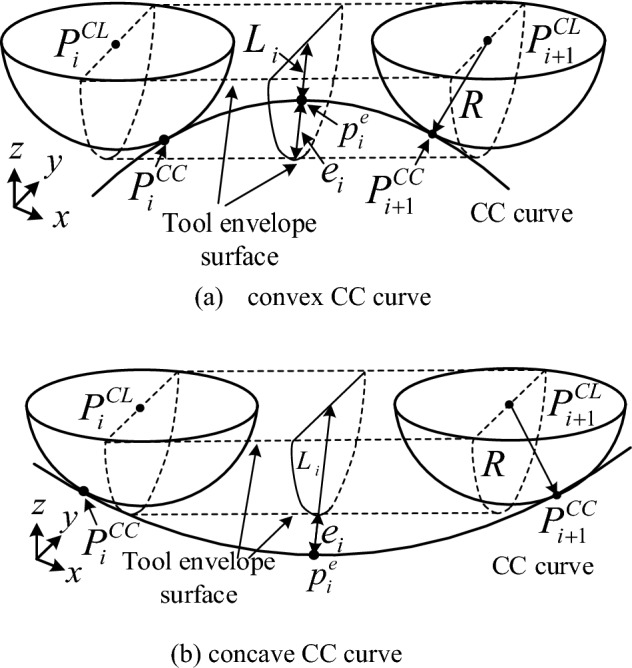
Step error $$e_{i}$$.

As shown in Fig. 1, the envelope formed by three-axis ball-end tool finishing machining is a cylinder with the line connecting two neighboring CL points as the axis. The step error $e_{i}$ between the CL line $P_{i}^{CL} P_{i + 1}^{CL}$ and CC curve can be expressed by Eq. (1), where $L_{i}$ is the three-dimensional distance from the point $p_{i}^{e}$ on the CC curve to the line $P_{i}^{CL} P_{i + 1}^{CL}$. When the CC curve is convex, $p_{i}^{e}$ is the point on the CC curve with the minimum distance to $P_{i}^{CL} P_{i + 1}^{CL}$. On the contrary, when CC curve is concave, $p_{i}^{e}$ is the point on the CC curve with the maximum distance to $P_{i}^{CL} P_{i + 1}^{CL}$.

1 $$ \left\{ \begin{gathered} e_{i} { = }\left| {R - L_{i} } \right| \hfill \\ L_{i} { = }\frac{{\left| {\overrightarrow {{P_{i}^{CL} p_{i}^{e} }} \times \overrightarrow {{P_{i}^{CL} P_{i + 1}^{CL} }} } \right|}}{{\left| {\overrightarrow {{P_{i}^{CL} P_{i + 1}^{CL} }} } \right|}} \hfill \\ \end{gathered} \right. $$

The geometric iteration methods are commonly used to calculate step error values. Zhao^1^ proposed to calculate the maximum chord error point on the CC curve by a golden section method. Min^2^ calculated the slope of the curve and the connecting line between two adjacent CC points, and the step error value is calculated by the distance formula at the point with the same slope. Our team^3^ uses discrete bottom circles of flat-end tool instead of tool envelope surface to calculate step error iteratively.

Step error is calculated by obtaining discrete points on the CC curve and iteratively calculating the maximum or minimum distance to the CC or CL segments^1–3^. However, these iterative methods cannot use previous calculating experience to calculate following step error. It is difficult for iterative methods to improve efficiency.

### Classical BP neural network

Artificial neural networks can use the "experience" gained from previous datasets to predict the outcomes of new datasets. As the most widely used neural network, BP neural network^4^ is a kind of mathematical model that simulates the neural system of human brain in order to handle complex information. BP neural networks with at least three layers can approximate any nonlinear function with arbitrary order of accuracy. BP neural network is capable of self-learning, self-adaption, robustness, and generalization. It has been widely applied in function approximation, pattern recognition and image processing^5^, and can even be used for stock price prediction^6^, software fault diagnosis^7^, signal processing^8^ and medical system^9^. In mechanical engineering, BP neural network is used to predict the relationship between tool rake angle, helix angle and machining deformation of thin-walled parts^10^, as well as the selection and optimization of process parameters for electro-discharge machining (EDM) with a flat electrode^11^.

**Figure 2:**
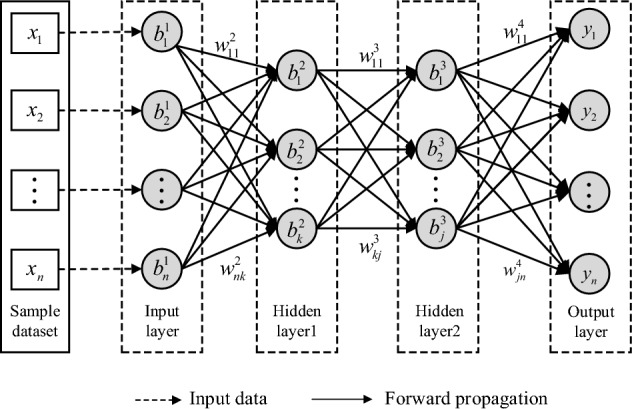
BP neural network with two hidden layers.

BP neural network consists of an input layer, some hidden layers and an output layer, as shown in Fig. 2. The input layer receives sample datasets. The hidden layers carry out the calculation based on the data of the input layer. The output layer outputs the calculated result of hidden layers. Every layer of BP neural network is made up of independent processing units known as neurons. All neurons in a layer connect to all neurons in the next layer^12^. Before data is passed to the neurons in the next layer, the neurons in this layer must be set weight, and this layer should also be set a bias. The weight represents the importance of a neuron, and a larger value indicates that a neuron has a greater impact on the outcome. The bias is used to make a final adjustment to the calculated values of a hidden or output layer, which can improve calculation accuracy.

A classical BP neural network iteration cycle consists of two parts, data forward propagation and error backpropagation. The data in the input layer is passed to hidden layers in forward propagation, and then weighted and summed over. Finally, the data is passed to the output layer. If the calculated value of the network is far from the expected value, their error needs to be back-propagated. In the process of error backpropagation, the weights between neurons are adjusted for smaller output error. The dataset is generally divided into a training set and a test set. They are used to train the network and verify its accuracy and generalization, respectively.

As shown in Fig. 2, in the forward propagation process, the weight between the first neurons in the input layer and the hidden layer 1 is $w_{11}^{2}$. *n* is the number of parameters in samples. *k* and *j* are the numbers of neurons in Hidden layer 1 and Hidden layer 2, respectively.

The weighted sum between the *j*th neuron and all neurons in the previous layer can be obtained by Eq. (2). In Eq. (2), *M* is the total number of neurons in the previous layer.$x_{i}$ is the value of the *i*th neuron in this layer, and $b_{k}$ is the bias of the layer. *h* is the serial number of layer where the *j*th neuron is located.

2 $$ S_{j}^{h} = \sum\limits_{i = 1}^{n} {w_{ij}^{h} } x_{i} + b_{j}^{h} $$

Because linear models have limited approximation capability, it is necessary for network to use a nonlinear function as activation function to improve expression capability. In 2011, ReLU function in Eq. (3) was demonstrated to further improve training of deep neural networks, which has strong biological and mathematical underpinning^13^.

3 $$ y_{j} = \max (0,S_{j}^{k} ) $$

After the output layer obtains the calculated value, a measure is required to determine the similar degree between the expected value $d_{j}$ and calculated value $y_{j}$. The Mean Square Error (MSE) function in Eq. (4) is commonly used as the loss function, where *n* is the total number of samples.

4 $$ E_{D} = \frac{1}{n}\sum\limits_{j = 1}^{n} {\left( {d_{j} - y_{j} } \right)^{2} } $$

A smaller $E_{D}$ in Eq. (4) means more accurate calculation. However, $E_{D}$ is too large to use in most cases. $E_{D}$ is an average value of the square sum, and also a multivariate quadratic function. In order to reduce the value of $E_{D}$, back propagation is required to calculate the gradient $\frac{{\partial E_{D} }}{{\partial w_{i} }}$. When $\frac{{\partial E_{D} }}{{\partial w_{i} }}$ tends to 0, all weighted values are the wanted results. The gradient descent formula for a classical BP neural network is shown in Eq. (5) and (6), where $\Delta w_{ij}^{k}$ is the adjusted amount and $\eta$ is the learning rate.

5 $$ w_{ij}^{k + 1} = w_{ij}^{k} + \Delta w_{ij}^{k} $$

6 $$ \Delta w_{ij}^{k} = - \eta \frac{{\partial E_{D} }}{{\partial w_{ij}^{k} }} $$

As a critical hyperparameter, the learning rate determines if the objective function can converge to a local minimum. Too high or too low learning rate of neural networks has a detrimental effect on the computational accuracy of the model^14^. A suitable learning rate can make the target function quickly restore the partial optimal solution. However, there is still no suitable formula to set the learning rate so far. Therefore, it should be set by users.

BP neural networks have high flexibility to adjust and optimize their construction based on the characteristics of step error. Various ways such as altering activation functions and the number of layers, neurons, can be employed to obtain a well-suited model for step error prediction.

In order to realize the application of BP neural networks to step error prediction and overcome BP neural network disadvantages such as the tendency to fall into local optimal solution, slow convergence, overfitting, etc.^5^, this paper focuses on creating BP neural network data samples, designing network struct and training network.

### Construction of this paper

In "Sample dataset design of BP neural network for step error prediction", based on the geometry principle of step error, the core parameters of step error calculation are obtained, and mapped to a same scale by Z-score normalization. This mapping can eliminate unfavorable effect of singular parameters. These parameters form the neural network's samples. In "BP neural network design and optimization for step error prediction", considering the feature that the theoretical value of step error is determined by a very small number of unknown key CC points on the curve, Dropout technique is added to shield a part of neurons during every iteration, which reduces the effect of redundant non-core CC points and improves the generalization ability of neural network. In the back propagation process, the SGDM optimizer is used in the gradient descent of the loss function. It helps the calculated values of neural network get out of the local optimal result region faster, which improves calculation accuracy. In "Algorithm implementation and validation", the neural network model is constructed, trained and verified.

## Sample dataset design of BP neural network for step error prediction

Before BP neural network predicts step error values, the network is firstly trained with plentiful sample dataset. The dataset should include all data closely related to step error.

“Geometry principle and calculation methods of step error” shows that step error is the minimum or maximum distance between a CL line and local CC curve. Not local CC curve but discrete points on it are usually used to calculate distances to the CL line, which is more convenient in practical application. Step error is calculated based on the discrete points and two CL points together with tool radius. These points and tool radius are input data, and the value of step error is the only output data, which form the dataset as shown in Table 1. $\left\{ {p_{j} } \right\}$ in Table 1 are the discrete points on the local CC curve, which are generally obtained by an iso-parametric method.

**Table 1:** The sample dataset for step error prediction.

Input layer			Output layer	Extra data
Points on CC curve	CL points	Tool radius	Output step error	Expected step error
$\left\{ {p_{j} } \right\}$,$P_{i}^{CC}$,$P_{i + 1}^{CC}$	$P_{i}^{CL}$,$P_{i + 1}^{CL}$	*R*	$e_{i}^{o}$	$e_{i}$

In neural network training process, errors between the expected and output step error values are essential for backpropagation. So, the dataset should include the expected value, i.e., the theoretical value.

## BP neural network design and optimization for step error prediction

The classical BP neural network probably overfits and gets stuck in a local optimal result in application^6^, which increase errors between the output and expected values. In this section, improvements on dataset normalization, hidden layer and network weight optimization are proposed to decrease the error.

### Dataset normalization and number determination of hidden layers and neurons

Table 1 shows that the dataset includes three kinds of parameters, coordinates of discrete points, tool radius and step error. In order to get rid of the effect of scale between parameters, it is necessary to map different data to a same scale. As shown in Eq. (7), the dataset is normalized by Z-score Normalization. $X_{{{\text{sc}}ale}}$ is a normalized value. *x* is a parameter to be normalized. $\mu$ and *S* are the mean error and standard deviation, respectively. After normalization, the dataset is assigned to the input layer and forward propagate in the following hidden layers by the weighted summation algorithm Eq. (2).

7 $$ \left\{ \begin{gathered} X_{{{\text{sc}}ale}} = \frac{x - \mu }{S} \hfill \\ \mu = \frac{1}{n}\sum\limits_{i = 1}^{n} {x_{i} } \hfill \\ S = \sqrt {\frac{1}{n - 1}\sum\limits_{i = 1}^{n} {\left( {v_{i} - \mu } \right)^{2} } } \hfill \\ \end{gathered} \right. $$

The number of neurons in the input layer are equal to the number of parameters in the dataset. The output layer has only one neuron to output step error value. The number of hidden layers and the number of neurons in every hidden layer are user-defined.

In classic BP neural network, more hidden layers and neurons means more calculation. So, it is critical to set as few hidden layers and neurons as possible when BP neural network can achieve accuracy need in step error prediction. Equation (8) and (9) are common formulas for calculating the number of neurons^15^. $n_{1}$ is the number of hidden layer neurons. *n* is the number of input layer neurons. *m* is the number of output layer neurons, and *a* is a constant between^1,10^.

8 $$ n_{1} = \sqrt {n + m} + a $$

9 $$ n_{1} = \log_{2} n $$

### Network construction and forward algorithm design based on Dropout technique

According to the introduction of geometric principle in “Geometry principle and calculation methods of step error”, the step error value is determined by a specific unknown point on the CC curve. The points in $\left\{ {p_{j} } \right\}$ are evenly distributed on the local CC curve between two adjacent CC points and most points are far away from this specific point. These points have almost no effect on the result and can be seen as irrelevant points. Decreasing the calculation of these unnecessary points can significantly improve the network’s efficiency.

**Figure 3:**
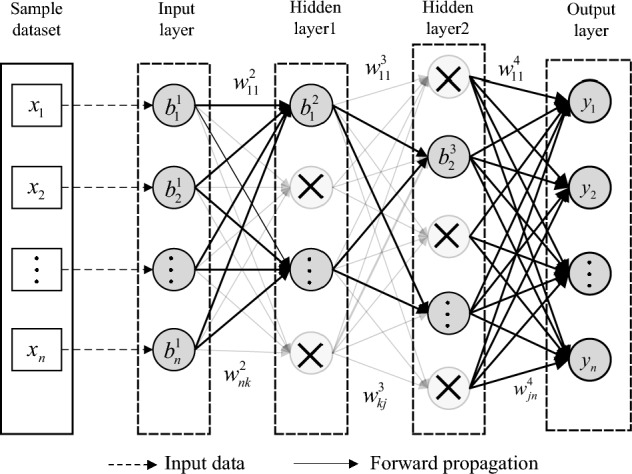
A dropout neural network.

The Dropout technique is firstly proposed by Hinton in 2012^16^. Neurons in hidden layers are drop out with a certain probability^17^. A dropout neural network is shown in Fig. 3. Compared with common network in Fig. 2. The dropout neural network contains less neurons and forward propagation.

When a hidden layer transfer “experience” to the next hidden layer, the Dropout technique can stop data of many irrelevant points from forward propagating, which makes every neuron in Dropout hidden layers more robust and improves both efficiency and accuracy^17,18^. Based on the Dropout technique and the methods in "Dataset normalization and number determination of hidden layers and neurons", the forward algorithm for step error prediction is proposed as following.

Step 1 Use Eq. (7) to normalize the dataset.

Step 2 Set the number of hidden layers and the neurons of the hidden layers.

Step 3 Import all the samples into the network.

Step 4 For every neuron in hidden and output layers, a weight value is set based on a normal distribution between any two neurons in adjacent layers, and a bias value is also set by a uniform distribution. A weight value matrix $w_{hj}$ and a bias value matrix $b_{ij}$ can be obtained as shown in Eqs. (10) and (11). *h* and *j* are the numbers of neurons in two adjacent layers. *i* is the number of samples. *c* and *d* are constants, respectively.

10 $$ w_{hj} = \left[ {\begin{array}{*{20}c} {w_{11} } & {w_{12} } & \cdots & {w_{1j} } \\ {w_{21} } & {w_{22} } & \cdots & {w_{2j} } \\ \vdots & \vdots & \cdots & \vdots \\ {w_{h1} } & {w_{h2} } & \cdots & {w_{hj} } \\ \end{array} } \right]\sim N\left( {\mu ,\sigma^{2} } \right) $$

11 $$ b_{ij} = \left[ {\begin{array}{*{20}c} {b_{11} } & {b_{12} } & \cdots & {b_{1j} } \\ {b_{21} } & {b_{22} } & \cdots & {b_{2j} } \\ \vdots & \vdots & \cdots & \vdots \\ {b_{i1} } & {b_{i1} } & \cdots & {b_{ij} } \\ \end{array} } \right]\sim U\left( {c,d} \right) $$

Step 5 Perform a weighted summation algorithm on the dataset by Eq. (12).

12 $$ \left[ {\begin{array}{*{20}c} {x_{11} } & {x_{12} } & \cdots & {x_{1h} } \\ {x_{21} } & {x_{22} } & \cdots & {x_{2h} } \\ \vdots & \vdots & \cdots & \vdots \\ {x_{i1} } & {x_{i2} } & \cdots & {x_{ih} } \\ \end{array} } \right]\left[ {\begin{array}{*{20}c} {w_{11} } & {w_{12} } & \cdots & {w_{1j} } \\ {w_{21} } & {w_{22} } & \cdots & {w_{2j} } \\ \vdots & \vdots & \cdots & \vdots \\ {w_{h1} } & {w_{h2} } & \cdots & {w_{hj} } \\ \end{array} } \right] + \left[ {\begin{array}{*{20}c} {b_{11} } & {b_{12} } & \cdots & {b_{1j} } \\ {b_{21} } & {b_{22} } & \cdots & {b_{2j} } \\ \vdots & \vdots & \cdots & \vdots \\ {b_{i1} } & {b_{i2} } & \cdots & {b_{ij} } \\ \end{array} } \right] = \left[ {\begin{array}{*{20}c} {y_{1}^{\prime } } \\ {y_{2}^{\prime } } \\ \vdots \\ {y_{i}^{\prime } } \\ \end{array} } \right] $$

Step 6 Use Dropout technique in every two hidden layers. The parameter *r* is set following Bernoulli distribution in Eq. (13).

13 $$ \left\{ {\begin{array}{*{20}l} {P\left( {r = 1} \right) = p} \hfill \\ {P(r = 0) = 1 - p} \hfill \\ \end{array} } \right.,0 < p < 1 $$

Then let $y_{i}^{\prime \prime } = ry_{i}^{\prime }$, and $y_{i}^{\prime }$ has a 1-*p* probability stop transmit data to the next neuron.

Step 7 Use Eq. (3) to calculate $y_{i}^{\prime \prime }$, and import the dataset to the next layer.

Step 8 Repeat Step 5, 6 and 7 until the last hidden layer.

Step 9 Use Step 7 to convey the dataset from the last hidden layer to the output layer.

Step 10 Calculate the error between the output value and the expect value.

### Network weight optimization based on SGDM optimizer in backpropagation

In BP neural network, if initial network weights are not set in accordance with the actual situation, calculated results are prone to fall into local optimal values. For free-form CC curve, the points corresponding to theoretical step errors are usually located in the midpoint neighborhood of CC curve. Therefore, the initial weights are usually set random numbers conforming to the normal distribution before forward propagation, and the weight values are adjusted in backpropagation process.

After thousands of iterations, the convergence speed of a classical BP neural network is greatly reduced. In most cases, a suitable optimizer is necessary to be used to improve the convergence speed and prediction accuracy. “Classical BP neural network” introduces the gradient descent algorithm in the backpropagation process. However, this algorithm needs to compute all the data in the dataset and costs a lot of time. Moreover, the calculation result obtained by this algorithm is prone to a local optimal value. Therefore, this section uses an optimizer to address these deficiencies.

As a widely used optimizer in neural networks, SGD (Stochastic Gradient Descent) optimizer one sample from the whole dataset at random into the gradient descent algorithm every time. Different from BGD (Batch Gradient Descent) optimizer that need to calculate the gradients of all samples^19^, only a part of samples are selected for gradient descent in SGD and can get close to global optimal, which improves calculation efficiency and accuracy.

The SGD optimizer performs better in complex nonlinear models, and can produce sparser values^20,21^. However, it introduces noise in the gradient^22^, and calculated results fluctuate around global optimal results. For this reason, much research has improved fitting accuracy of BP neural networks by optimizing the momentum^23,24^. In the tool path of a free-form surface, the variation of curvature is continuous. However, the addition of the SGD optimizer and momentum can make the gradient descent smoother during the neural network training process, and enhance model stability. So, the SGD optimizer and momentum term are added to the model training in this paper.

Momentum is the weighted sum of all the previous gradient. It can make the process of gradient descent more stable. Therefore, momentum is added to the SGD optimizer in this section. The weight adjustment formula based on the SGDM (Stochastic Gradient Descent with Momentum) optimizer is shown in Eq. (14). $v_{t + 1}$ is the momentum. *t* is the number of current iterations. $t = 0$ means the first iteration, and the momentum $v_{0} = 0$. $\beta$ is the momentum hyperparameter and should be set in the range (0,1). It determines how much the gradient of the previous iteration affects the direction of the current gradient. $\eta$ is the learning rate of the network.

14 $$ \left\{ {\begin{array}{*{20}c} {w\left( {t + 1} \right) = w\left( t \right) + v_{t + 1} } \\ {v_{t + 1} = \beta v_{t} - \eta \frac{{\partial E_{D} }}{\partial w\left( t \right)}} \\ \end{array} } \right. $$

In Eq. (14), the weight $w\left( t \right)$ of the past iterations has an impact on the current one $w\left( {t + 1} \right)$. The gradient of the *t* + 1th iteration is the weighted sum of the previous all *t* gradients, which incorporate gradient descent from past iterations. The earlier iterations have less impact on the change of the current weight.

The addition of momentum can make the calculation result rush out of the local optimal value by "inertia" in the process of gradient descent. This optimizer takes advantage of "inertia effect" to suppress oscillation during training, and ensures gradient stability in the current iteration. The SGDM optimizer can promote calculation results closer to global optimal results. The gradient decreases more smoothly and is closer to the extreme value of gradients^25^.

## Algorithm implementation and validation

### Network construction

#### (1) Sample construction

**Figure 4:**
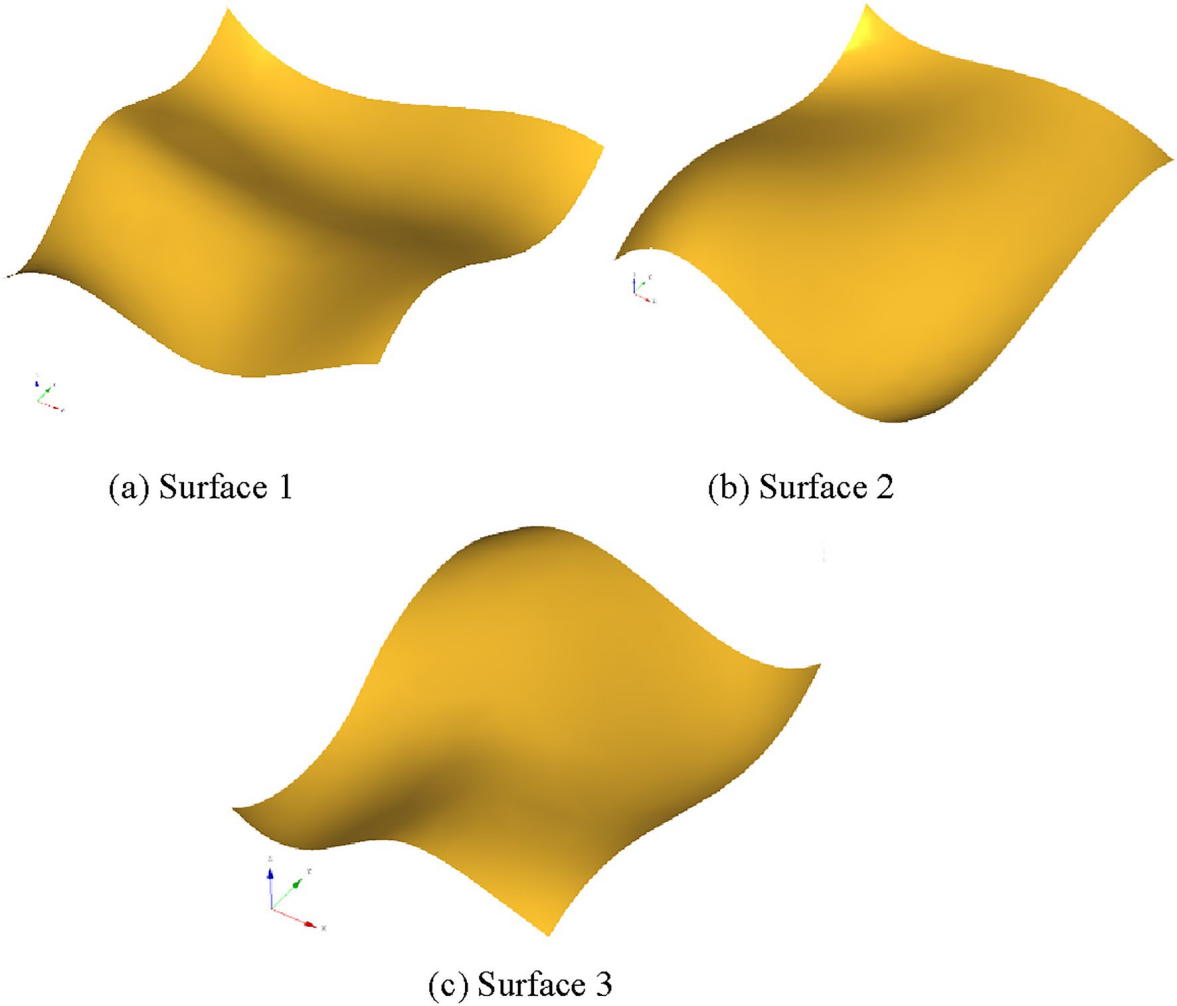
Three free-form surface models.

Three free-form surface models (shown in Fig. 4 with the bounding box size of $120\quad {\text{mm}} \times 140\quad {\text{mm}} \times 38\quad {\text{mm}}$,$135\quad {\text{mm}} \times 175\quad {\text{mm}} \times 68.28\quad {\text{mm}}$,and $150\quad {\text{mm}} \times 150\quad {\text{mm}} \times 54.02\quad {\text{mm}}$) are used to generate tool path and obtain the sample data. Table 1 shows a sample structure, where the tool radius is 5 mm.

#### (2) Network parameter determination

The neural network model in this paper contains one input layer, one output layer, and two hidden layers. According to Eq. (8), there are 28 neurons in the first hidden layer and 20 neurons in the second hidden layer. The relationship between the probability *p* and MSE loss function is shown in Table 2. When *p* = 0.5, the MSE value is the minimum. Therefore, *p* is set 0.5.

**Table 2:** *p* and MSE.

*P*	0.3	0.4	0.5	0.6	0.7	0.8	0.9
MSE	0.011	0.009	0.004	0.006	0.0053	0.017	0.021

As shown in "Network weight optimization based on SGDM optimizer in backpropagation",$w_{i}$,$\eta$ and $\beta$ should be set before forward propagation. The weight values in every layer are set random values that conform to a normal distribution before forward propagation. The mean and the standard deviation of this normal distribution are 0 and 1, i.e., $X\sim N\left( {0,1} \right)$. The learning rate $\eta$ determines the step size of the gradient descent at every iteration and whether the loss function can converge to the minimum value. A larger learning rate causes faster gradient descent. But it will also lead to a decrease in computational accuracy. $\beta$ represents influence degree of past weights on the current gradient. A larger $\beta$ means greater influence. In order to satisfy calculation efficiency and accuracy, after several tests and adjustments to the values of $\eta$ and $\beta$, we set $\eta = 1.0 \times 10^{ - 5} ,\beta = 0.8$.

### Train network on surface 1 and obtain initial neural network model for step error prediction

Two hundred lines of iso-parametric tool path are planned for Surface 1. Every tool path contains 100 CL points and 19,800 samples are generated. The range of step error values is $\left[ {0.01\quad {\mu m},13\quad {\mu m}} \right]$. 70% of the samples are randomly chosen as a training set for the step error prediction network, while the remaining 30% samples are used as the test set.

**Figure 5:**
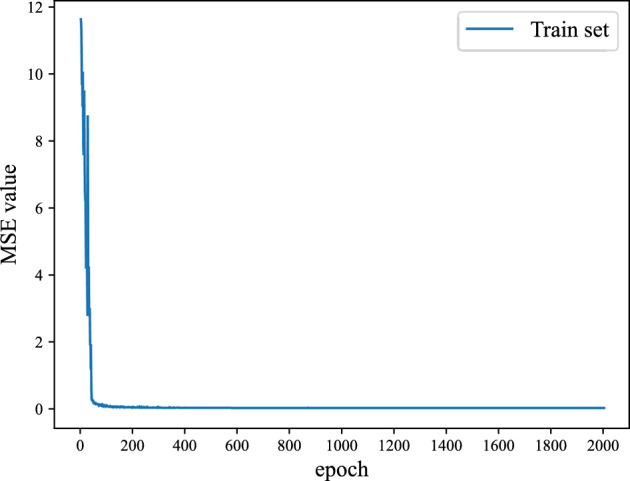
MSE loss value change diagram of Surface 1.

As shown in Fig. 5, When the iteration count is less than 50, there are significant differences between the actual value and predicted value, which causes great MSE values. As the iteration count increases, the MSE values gradually decreases and stabilizes at 0.001 after 2000 epochs. So, the network training ends at 2000th epoch and the trained network is used as the initial neural network for following step error prediction.

**Figure 6:**
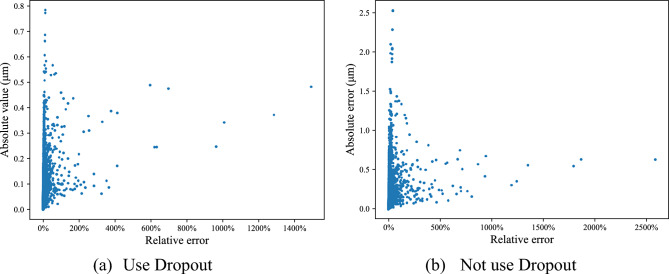
Step error between theoretic values and predicted values in Surface 1.

In "Network construction and forward algorithm design based on dropout technique", the Dropout technique is Applied in the initial neural network training. The errors between theoretical and predicted step error values are illustrated in Fig. 6a. As a comparison, the neural network is trained without Dropout technique and the errors is illustrated in Fig. 6b. Obviously, the overall error with Dropout technique is lower than that without Dropout, which verifies the effectiveness of Dropout technique.

The detail data of the errors in Fig. 6a is shown in Table 3. Only 10.33% of relative errors are greater than 10%, but 85.97% of their theoretical values are less than 1 μm. The maximum of all absolute error values is 0.78 μm, but 99.71% values are less than 0.5 μm. Therefore, the neural network can meet the accuracy requirement of practical application.

**Table 3:** Prediction error of test samples.

(a) Absolute error						
Absolute error (μm)	< 0.1	0.1–0.2	0.2–0.3	0.3–0.4	0.4–0.5	0.5–0.8
Number	3616	1629	440	206	32	17
Ratio (%)	60.88	27.42	7.41	3.47	0.54	0.29

(b) Relative error						
Relative error	< 1%	1–5%	5–10%	10–15%	15–20%	> 20%
Number	1190	3363	774	187	109	317
Ratio (%)	20.03	56.62	13.03	3.15	1.84	5.34

### Predict step error values of Surface 2

200 lines iso-parametric tool path with 100 CL points per row are generated for Surface 2. The sample dataset including 19,800 samples as same as Surface 1. All the step error values are in $\left[ {0.01\;{\mu m},14\;{\mu m}} \right]$. To comprehensively evaluate the network, five different ratio 0%, 1%, 5%, 10%, 15% samples are used to train the network.

**Figure 7:**
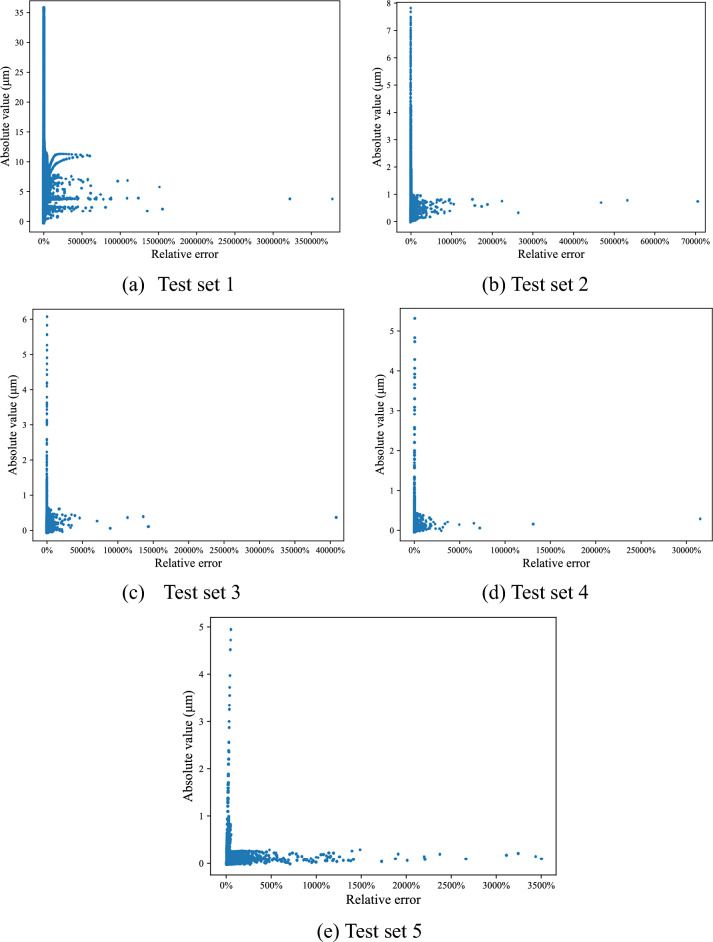
Step error between theoretic values and predicted values in Surface 2.

Firstly, the initial neural network obtained in "Train network on Surface 1 and obtain initial neural network model for step error prediction" is not trained with samples of Surface 2 and predict directly all samples of Surface 2. Subsequently, as shown in Table 4, 1%, 5%, 10%, 15% of all samples are randomly selected to train the initial neural network and obtain an improved network, separately. Five networks predict the remaining samples, separately. The results are illustrated in Fig. 7.

**Table 4:** Test sets for surface 2.

Test set	1	2	3	4	5
Extract sample proportion	0%	1%	5%	10%	15%
The number of samples in test set	19,800	19,602	18,810	17,820	16,830

As more samples are used to train the network, the prediction accuracy of the remaining samples improved continuously. Specifically, the absolute error maximum decreases from 35.21 to 5.25 μm, while the proportion of absolute error values less than 1 μm increases from 11.17 to 99.80%.

When 15% of the samples are used to train the network, absolute error values of remaining samples are all less than 1 μm. 78.28% of the samples have relative error values less than 10%. Thus, the neural network with the highest prediction accuracy is selected as the improved neural network model for following testing.

### Predict step error values of Surface 3

**Figure 8:**
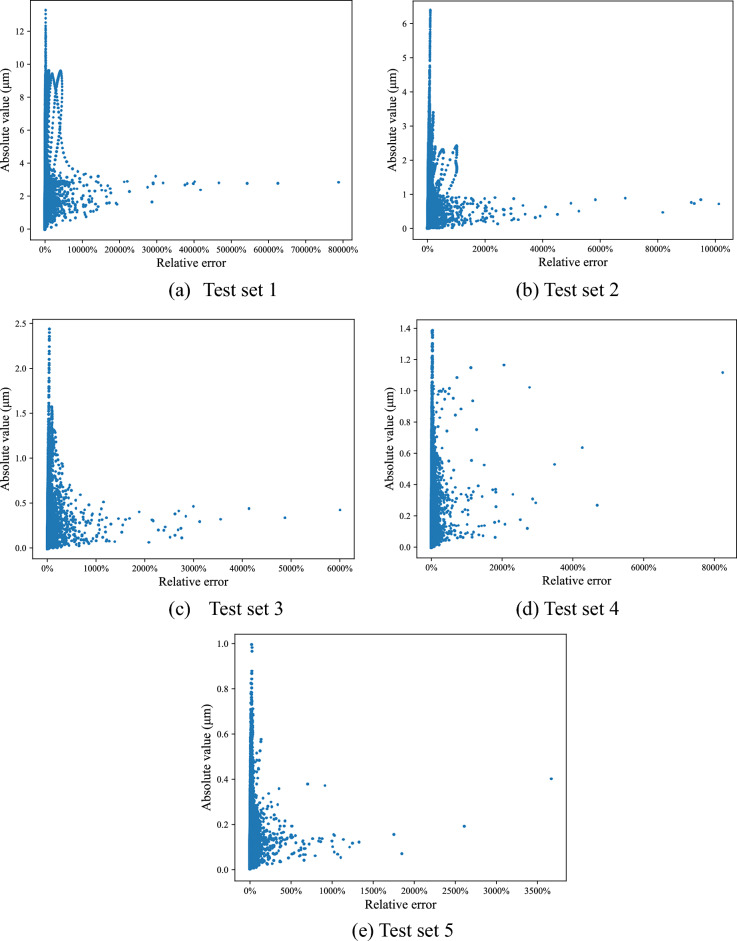
Step error between theoretic values and predicted values in Surface 3.

Same with Surface 2, a sample dataset of 19,800 samples is planned for surface 3, as shown in Table 5. All the step error values are in $\left[ {0.01\;{\mu m},9\;{\mu m}} \right]$. After trained by 15% of the samples from Surface 2 and all samples from Surface 1, the improved network is used to test samples of Surface 3. The testing process is same with that used in surface 2, and the testing set is presented in Table 6. The absolute and relative errors of the test results are shown in Fig. 8. 0%, 1%, 5%, 10%, 15% of samples are used to train the network, separately. The absolute error maximum of Surface 3 decreases from 13.21 to 0.99 μm. The proportion of absolute error values less than 1 μm increases from 31.54 to 100%, which confirms that the more training samples, the greater prediction accuracy.

**Table 5:** Test sets for surface 3.

Test set	1	2	3	4	5
Extract sample proportion	0%	1%	5%	10%	15%
The number of samples in test set	19,800	19,602	18,810	17,820	16,830

**Table 6:** Prediction error of Surface 2 test set.

(a) Absolute error						
Absolute error	< 0.5 μm	0.5–1 μm	1–2 μm	2–3 μm	3–4 μm	4–5 μm
Number	16,647	151	16	6	7	3
Ratio (%)	98.90	0.90	0.01	0.04	0.04	0.01

(b) Relative error						
Relative error	< 1%	1–5%	5–10%	10–15%	15–20%	> 20%
Number	4011	7059	2105	1035	748	1872
Ratio (%)	23.83	41.94	12.51	6.15	4.44	11.12

Comparing the prediction results of Surfaces 2 and 3 in Tables 6 and 7, it can be observed that the proportion of samples with an absolute error less than 0.4 μm increased from 92.38 to 98.32%. Furthermore, the average absolute error decreased from 0.17 to 0.10 μm, while the proportion of samples with a relative error less than 10% increased from 78.28 to 83.63%. Thus, the conclusion can be drawn that the more samples the model receives during training, the higher the prediction accuracy.

**Table 7:** Prediction error of Surface 3 test set.

(a) Absolute error						
Absolute error	< 0.1 μm	0.1–0.3 μm	0.3–0.5 μm	0.5–0.7 μm	0.7–1 μm	
Number	10,587	5417	635	159	32	
Ratio (%)	62.91	32.19	3.77	0.94	0.19	

(b) Relative error						
Relative error	< 1%	1–5%	5–10%	10–15%	15–20%	> 20%
Number	3112	7662	2627	1125	632	1672
Ratio (%)	18.49	45.53	19.61	5.68	2.76	7.93

Finally, by comparing the computation time required to calculate step errors of surfaces 2 and 3 using the geometric method^26^ and the prediction time for the testing set based on the model proposed in this paper (including the time for importing the model), as shown in Table 8, the results indicate a significant improvement in prediction efficiency using the proposed model compared to the geometric method. The computer used is i7-12700KF with 32 GB RAM, NVIDIA 3070Ti, the neural network framework is PyTorch, and the Python version is 3.9.

**Table 8:** Calculation Time Table.

Method	Surface 2 (s)	Surface 3 (s)
Geometric method	9.267	9.761
Proposed method	2.861	2.798

## Summary

Currently, geometric iteration methods are the most common methods of calculating step error, and cost more computation time for higher precision. In order to improve computational efficiency with required accuracy, this paper proposes a new step error prediction method based on BP neural networks, which can be trained on GPUs. Core parameters required for step error calculation are taken as data samples for the neural network, and Dropout technique is employed in the neural network construction for preventing overfitting. A SGDM optimizer is added to back propagation in network training to improve the accuracy and stability of step error prediction.

The prediction results of three surfaces show more training samples make the prediction more accurate. After the existed network is trained by 15% of samples from new surface, the predicted values of the remaining samples have errors less than 1 μm, which can meet practical application. The computation time is only one-third of the traditional geometric method. All the results verify the effectiveness and efficiency of this method. This study provides technical support for using neural networks to calculate geometric errors in NC machining.

In future research, methods of improving neural network prediction accuracy can be further explored and extended to other NC machining geometric error prediction.

## Data Availability

Main data generated or analyzed during this study are included in this article. All data in this article are available from the corresponding author on reasonable request.
